# Supplementary material for: Combined severe-to-profound hearing and vision impairment—Experiences of daily life and need of support, an interview study
Source: PLoS One. 2023 Jun 15;18(6):e0280709. doi: 10.1371/journal.pone.0280709 (PMC10270357; doi:10.1371/journal.pone.0280709)
Supplement: S1 File — (PDF) [file pone.0280709.s001.pdf]

Interview Guide

Background information
Would you tell us a little about yourself, who you are and what you do daily etc.?

Open semi-structured questions

Question Area 1: *Vision loss and hearing impairment, their duration and possible effect on daily life.*

1. How long have you had your combined vision loss and hearing impairment (from birth/childhood/acquired)?

2. Can you tell us about a typical day in your life? Please include what it looks like at work/school (if current) and in your spare time? (For instance, associations, contact with The Swedish Association of Hard of Hearing People (HRF) etc.)

3. Can you tell us more about, how your combined vision loss and hearing impairment affect your everyday life? **a)** Education/workplace/pension **b)** Social network (family, friends, co-workers etc.) and **c)** Support from the environment (society, neighbors etc.)

Question Area 2: *Limitations, connected to the combined vision loss and hearing impairment, regarding daily activities and the physical environment.*

4. Do you experience any limitations, connected to your combined vision loss and hearing impairment, in your performance of daily activities? Describe a few situations.

5. Do you experience any limitations, connected to your combined vision loss and hearing impairment, in the physical environment that you on a day-to-day basis find yourself in? Describe a few situations.

6. Do you have any need of any form of personal support to ease/facilitate your everyday life?

7. Describe one or more situations that are affected by your combined vision loss and hearing impairment.
    a. Have these situations changed today compared to when you got your diagnosis?
    b. How do you handle situations on a day-to-day basis that are affected by your combined vision loss and hearing impairment? Do you use any specific strategies?
    c. Have you at any point tried to get help to handle situations that are affected by your combined vision loss and hearing impairment? (from birth/ /childhood/acquired)
    d. How have you handled difficulties connected to your combined vision loss and hearing impairment? What has helped you? Do you have any advice you would like to give others?

Question Area 3: *Availability*

8. How do you experience the possibility to take part of information provided by the society?

9. Do you have access to a deafblind interpreter? If 'yes' – do you feel that it aids you in your participation in the society?

Question Area 4: *Aid & support connected to combined vision loss and hearing impairment.*

10. Do you use hearing aids or cochlear implants? Please tell us how you feel they are supporting/aiding you?

11. Do you use any other technical aids at home or at work/school? Please tell us how you feel they are supporting/aiding you?

12. What is your experience towards the support from Hearing health care? Including Alternative Telephony.
    a) Do you feel like there is anything missing?
    b) Do you feel like anything has changed over time?
    c) Do you have any suggestions for improvement/more efforts?

13. How do you experience the support from Low Vision Centre? Including the Councelling and Support team for persons with deafblindness (Deafblind-team unit).
    a) Do you feel like there is anything missing?
    b) Do you feel like anything has changed over time?
    c) Do you have any suggestions for improvement/more efforts?

Finishing questions:

14. Which are your thoughts in the near future linked to your combined vision loss and hearing impairment?

15. How did you experience the questions in this interview?

16. Is there anything else you would like to add beyond what we have included in this interview?

Thank you for your participation! This is of great importance to improve our work and to give better care to patients with serious hearing impairment in combination with serious vision loss.

You are always welcome to call or email afterwards if you have any questions. Send an email to satu.turunen-taheri@ki.se or call 070 3682771.

Authors: Satu Turunen-Taheri, Annica Hagerman Sirelius, Åsa Skjönsberg, Sten Hellström och Gunnel Backenroth
