# Supplementary material for: Combined severe-to-profound hearing and vision impairment—Experiences of daily life and need of support, an interview study
Source: PLoS One. 2023 Jun 15;18(6):e0280709. doi: 10.1371/journal.pone.0280709 (PMC10270357; doi:10.1371/journal.pone.0280709)
Supplement: S2 File — (PDF) [file pone.0280709.s002.pdf]

Karolinska Universitetssjukhuset
Karolinska Institutet
Dept of Audiology and Neurotology
The Unit for Hearing Rehabilitation of Adults

2017-09-07

Would you like to participate in a study and improve the hearing care!

An interview study will be performed with the purpose to improve the hearing care.

The purpose of the interview study

The study will focus on patients with severe-to-profound hearing impairment in combination with severe vision loss. The overall purpose is to describe experiences and factors that affect daily life, especially everything including communication, and also which kind of support that has been given from the extended rehabilitation.

Where and how are the interviews being made?

The interviews will take place at the dept of Audiology and Neurotology, Tideliusgatan 12, floor 9, Rosenlund, Karolinska University Hospital, Stockholm. We can also consider conducting the interview at another location if that suits you better. The time will be booked when it suits you. The interview will take around one (1) hour. The interview will be recorded to support the memory of the interviewers.

Presentation of data

All research results are treated confidentially and the professionals involved in the study has absolute secrecy.

The results will be presented on a group level and there is no way to identifying you as an individual patient. The recorder interviews will be deleted after having completed the study.

141 86 Stockholm KAROLINSKA UNIVERSITETSSJUKHUSET HUDDINGE 08 - 585 815 65
171 76 Stockholm KAROLINSKA UNIVERSITETSSJUKHUSET SOLNA 08 - 5177 69 76 Reception
118 50 Stockholm KAROLINSKA UNIVERSITETSSJUKHUSET ROSENLUND 08 - 616 64 50

KAROLINSKA
Universitetssjukhuset

Participation is voluntary

We hope that you would like to participate. You can at any point cancel your participation without excuse and without it will affect your future care. If you give your consent for participation, please sign your signature down below. If you have any questions or need help to answer the form, feel free to send an email to satu.turunen-taheri@sll.se or call 070 368 2771.

The interviews is a part of a thesis work for PhD-thesis work for leg audiologist Satu Turunen-Taheri.

If you would like to participate in the study please fill out the form down below with your name and social security number and hand it over to your audiologist.

Sincerely

Satu Turunen-Taheri, PhD, MSc, leg audiologist

✂ ____

I hereby confirm ………………………………………………………… with social security number ………………………………… that I have been informed about the study and taken part in above information. I am aware of that my participation is voluntary and can at any point be cancelled without explanation and that it won’t affect my future care.

____

City and date Signature of the participant
