# Supplementary material for: Combined severe-to-profound hearing and vision impairment—Experiences of daily life and need of support, an interview study
Source: PLoS One. 2023 Jun 15;18(6):e0280709. doi: 10.1371/journal.pone.0280709 (PMC10270357; doi:10.1371/journal.pone.0280709)
Supplement: S3 File — (PDF) [file pone.0280709.s003.pdf]

KAROLINSKA
Universitetssjukhuset
Hörsel- och Balanskliniken
Enheten för Hörselrehabilitering Vuxna

2019-10-09

Karolinska Institutet

Intervjustudien som du deltagit i

Du har deltagit i en intervjustudie under 2018-2019 med övergripande syftet att beskriva erfarenheter och faktorer som påverkar det dagliga livet när man har en grav hörselnedsättning i kombination med grav synnedsättning, samt vilket stöd man fått från rehabiliteringen.

Nu är alla intervjuerna klara - transkriberade från de inspelade intervjuerna - och vi arbetar med analyserna och författandet av den kommande artikeln. Jag skickar härmed den transkriberade texten till Dig som avser Din intervju. Jag kommer även skicka den via mejl till de intervjudeltagare där jag har en e-post-adress.

Redovisning av data

Som vi nämnde vid intervjun så behandlas alla resultat konfidentiellt dvs ingen obehörig har möjlighet att få tillgång till materialet och deltagandet i intervjustudien kommer att bearbetas under sekretess. De inspelade intervjuerna kommer att raderas efteråt.

Ett till samtycke behövs

Enligt de etiska riktlinjerna behöver vi ett till samtycke från Dig som gäller för att vi ska kunna använda oss av citat från intervjun i den kommande artikeln respektive uppsatsen. Vi kommer givetvis avidentifiera de citaten så att det inte går att härleda citaten till vem som sagt vad.

Om du ger Ditt samtycke till att vi kan använda oss av några citat från intervjun, var god fyll i talongen nedan med din namnunderskrift. Namn och personnummer är ifyllt redan. Skicka den tillbaka i det medföljande, portofria kuvertet.

Tack på förhand!

141 86 Stockholm KAROLINSKA UNIVERSITETSSJUKHUSET HUDDINGE 08 - 585 815 65
171 76 Stockholm KAROLINSKA UNIVERSITETSSJUKHUSET SOLNA 08 - 5177 69 76 Reception
118 50 Stockholm KAROLINSKA UNIVERSITETSSJUKHUSET ROSENLUND 08 - 616 64 50

Hörsel- och Balanskliniken
Enheten för Hörselrehabilitering Vuxna

Härmed bekräftar jag

………………………………………………… med

personnummer ………………………………… att jag godkänner att

man får använda sig utav ett antal citat från intervjun jag deltagit i.

Ort och datum

Underskrift av deltagaren

Har Du några frågor kring detta, går det bra att skicka e-post till satu.turunen-taheri@sll.se eller på mobilnummer 070 368 2771.

Med vänliga hälsningar

Satu Turunen-Taheri, doktorand, MSc, leg audionom
